# 3-(4-Fluoro­phen­yl)-1-(4-methoxy­phen­yl)prop-2-en-1-one

**DOI:** 10.1107/S1600536809018121

**Published:** 2009-05-29

**Authors:** Pu-Su Zhao, Xian Wang, Huan-Mei Guo, Fang-Fang Jian

**Affiliations:** a New Materials and Function Coordination Chemistry Laboratory, Qingdao University of Science and Technology, Qingdao 266042, People’s Republic of China; b Microscale Science Institute, Weifang University, Weifang 261061, People’s Republic of China

## Abstract

The title compound, C_16_H_13_FO_2_, was prepared from 4-methoxy­hypnone and 4-fluoro­benzophenone by Claisen–Schmidt condensation. All the bond lengths and bond angles are in normal ranges. The dihedral angle formed by the two benzene rings is 33.49 (2)°. The crystal packing is stabilized by inter­molecular C—H⋯O hydrogen-bonding inter­actions.

## Related literature

For the biological activity of chalcones, see: Hsieh *et al.* (1998 ▶); Anto *et al.* (1994 ▶). For the effectiveness of chalcones against cancer, see: De Vincenzo *et al.* (2000 ▶); Dimmock *et al.* (1998 ▶). For a related structure, see: Guo *et al.* (2008 ▶).
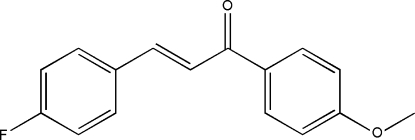


## Experimental

### 

#### Crystal data

C_16_H_13_FO_2_
*M* *_r_* = 256.26
Orthorhombic, 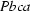

*a* = 7.457 (4) Å
*b* = 11.072 (6) Å
*c* = 31.063 (18) Å
*V* = 2565 (3) Å^3^
*Z* = 8
Mo *K*α radiation
μ = 0.10 mm^−1^
*T* = 273 K
0.13 × 0.12 × 0.09 mm

#### Data collection

Bruker SMART CCD area-detector diffractometer
Absorption correction: none
14904 measured reflections
3050 independent reflections
2203 reflections with *I* > 2σ(*I*)
*R*_int_ = 0.039

#### Refinement

*R*[*F* ^2^ > 2σ(*F* ^2^)] = 0.039
*wR*(*F* ^2^) = 0.116
*S* = 1.01
3050 reflections
173 parameters
H-atom parameters constrained
Δρ_max_ = 0.17 e Å^−3^
Δρ_min_ = −0.12 e Å^−3^

### 

Data collection: *SMART* (Bruker, 1997 ▶); cell refinement: *SAINT* (Bruker, 1997 ▶); data reduction: *SAINT*; program(s) used to solve structure: *SHELXS97* (Sheldrick, 2008 ▶); program(s) used to refine structure: *SHELXL97* (Sheldrick, 2008 ▶); molecular graphics: *SHELXTL* (Sheldrick, 2008 ▶); software used to prepare material for publication: *SHELXTL*.

## Supplementary Material

Crystal structure: contains datablocks global, I. DOI: 10.1107/S1600536809018121/at2773sup1.cif

Structure factors: contains datablocks I. DOI: 10.1107/S1600536809018121/at2773Isup2.hkl

Additional supplementary materials:  crystallographic information; 3D view; checkCIF report

## Figures and Tables

**Table 1:** Hydrogen-bond geometry (Å, °)

*D*—H⋯*A*	*D*—H	H⋯*A*	*D*⋯*A*	*D*—H⋯*A*
C11—H11*A*⋯O2^i^	0.93	2.50	3.376 (2)	158

Symmetry code: (i) 
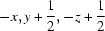
.

**Fig. 1.**
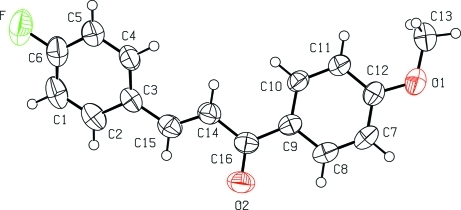
The molecular structure of the title compound with the atom-labeling scheme. Displacement ellipsoids are drawn at the 50% probability level.

**Fig. 2.**
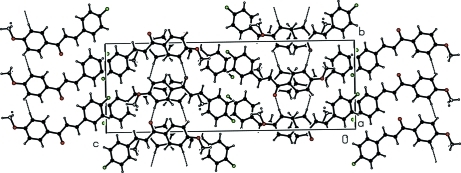
The packing and the hydrogen bonding interactions of (I),viewed down a axis.

## supplementary crystallographic information

### Comment

Among flavonoids, chalcones have been identified as interesting compounds having multiple biological actions which include antiinflammatory (Hsieh *et al.*, 1998) and antioxidant (Anto *et al.*, 1994). Of particular interest, the effectiveness of chalcones againist cancer has been investigated (De Vincenzo *et al.*, 2000; Dimmock *et al.*, 1998). As part of our search for new biologically active compounds we synthesized the title compound (I) and report its crystal structure here.

In the crystal structure of compound (I) (Fig. 1), the dihedral angle formed by the benzene rings (C1–C6) and (C7–C12) is 33.49 (2)°. All the bond lengths and bond angles are in normal ranges. (Guo *et al.*, 2008). There are intra- and intermolecular C—H···O hydrogen-bond interactions to stabilize the crystal structure (Table 1, Fig. 2).

### Experimental

A mixture of the 4-methoxyhypnone (0.02 mol), 4-fluorobenzophenone (0.02 mol) and 10% NaOH (10 ml) was stirred in ethanol (30 ml) for 3 h to afford the title compound (yield 83%). Single crystals suitable for X-ray measurements were obtailed by recrystallization from ethanol at room temperature.

### Refinement

H atoms were positioned geometrically and allowed to ride on their parent atoms, with C—H = 0.93–0.96 Å, respectively, and with *U*_iso_(H) = 1.2 or 1.5*U*_eq_ of the parent atoms.

### Crystal data

**Table:**

C_16_H_13_FO_2_	*F*(000) = 1072
*M**_r_* = 256.26	*D*_x_ = 1.327 Mg m^−^^3^*D*_m_ = 1.327 Mg m^−^^3^*D*_m_ measured by not measured
Orthorhombic, *P**b**c**a*	Mo *K*α radiation, λ = 0.71073 Å
Hall symbol: -P 2ac 2ab	Cell parameters from 4576 reflections
*a* = 7.457 (4) Å	θ = 2.6–27.2°
*b* = 11.072 (6) Å	µ = 0.10 mm^−^^1^
*c* = 31.063 (18) Å	*T* = 273 K
*V* = 2565 (3) Å^3^	Bar, yellow
*Z* = 8	0.13 × 0.12 × 0.09 mm

### Data collection

**Table:**

Bruker SMART CCD area-detector diffractometer	2203 reflections with *I* > 2σ(*I*)
Radiation source: fine-focus sealed tube	*R*_int_ = 0.039
graphite	θ_max_ = 28.1°, θ_min_ = 2.6°
φ and ω scans	*h* = −8→9
14904 measured reflections	*k* = −14→14
3050 independent reflections	*l* = −31→40

### Refinement

**Table:**

Refinement on *F*^2^	Secondary atom site location: difference Fourier map
Least-squares matrix: full	Hydrogen site location: inferred from neighbouring sites
*R*[*F*^2^ > 2σ(*F*^2^)] = 0.039	H-atom parameters constrained
*wR*(*F*^2^) = 0.116	*w* = 1/[σ^2^(*F*_o_^2^) + (0.0529*P*)^2^ + 0.3952*P*] where *P* = (*F*_o_^2^ + 2*F*_c_^2^)/3
*S* = 1.01	(Δ/σ)_max_ = 0.001
3050 reflections	Δρ_max_ = 0.17 e Å^−^^3^
173 parameters	Δρ_min_ = −0.12 e Å^−^^3^
0 restraints	Extinction correction: *SHELXL97* (Sheldrick, 2008), Fc^*^=kFc[1+0.001xFc^2^λ^3^/sin(2θ)]^-1/4^
Primary atom site location: structure-invariant direct methods	Extinction coefficient: 0.0151 (14)

### Special details

**Table:**

Geometry. All e.s.d.'s (except the e.s.d. in the dihedral angle between two l.s. planes) are estimated using the full covariance matrix. The cell e.s.d.'s are taken into account individually in the estimation of e.s.d.'s in distances, angles and torsion angles; correlations between e.s.d.'s in cell parameters are only used when they are defined by crystal symmetry. An approximate (isotropic) treatment of cell e.s.d.'s is used for estimating e.s.d.'s involving l.s. planes.
Refinement. Refinement of *F*^2^ against ALL reflections. The weighted *R*-factor *wR* and goodness of fit *S* are based on *F*^2^, conventional *R*-factors *R* are based on *F*, with *F* set to zero for negative *F*^2^. The threshold expression of *F*^2^ > σ(*F*^2^) is used only for calculating *R*-factors(gt) *etc*. and is not relevant to the choice of reflections for refinement. *R*-factors based on *F*^2^ are statistically about twice as large as those based on *F*, and *R*- factors based on ALL data will be even larger.

### Fractional atomic coordinates and isotropic or equivalent isotropic displacement parameters (Å^2^)

**Table:**

	*x*	*y*	*z*	*U*_iso_*/*U*_eq_	
O2	0.19197 (16)	0.39463 (9)	0.32285 (3)	0.0679 (3)	
O1	0.09858 (15)	0.57395 (10)	0.13185 (3)	0.0674 (3)	
C16	0.15026 (17)	0.49647 (12)	0.31027 (4)	0.0497 (3)	
C9	0.13654 (16)	0.52293 (11)	0.26375 (4)	0.0447 (3)	
C15	0.06589 (19)	0.56911 (13)	0.38189 (5)	0.0567 (4)	
H15A	0.0689	0.4883	0.3900	0.068*	
C10	0.07845 (18)	0.63325 (11)	0.24765 (4)	0.0484 (3)	
H10A	0.0485	0.6945	0.2668	0.058*	
C12	0.10839 (18)	0.56345 (12)	0.17528 (4)	0.0504 (3)	
F	−0.15021 (16)	0.89082 (12)	0.50973 (3)	0.1015 (4)	
C14	0.10856 (19)	0.59300 (12)	0.34157 (4)	0.0535 (3)	
H14A	0.1125	0.6731	0.3326	0.064*	
C11	0.06382 (18)	0.65460 (12)	0.20395 (4)	0.0498 (3)	
H11A	0.0246	0.7292	0.1939	0.060*	
C4	0.0339 (2)	0.77956 (13)	0.41030 (4)	0.0578 (4)	
H4A	0.0854	0.8098	0.3852	0.069*	
C8	0.17978 (19)	0.43239 (12)	0.23404 (5)	0.0550 (4)	
H8A	0.2185	0.3575	0.2439	0.066*	
C7	0.1660 (2)	0.45230 (13)	0.19088 (5)	0.0600 (4)	
H7A	0.1953	0.3910	0.1717	0.072*	
C3	0.01471 (18)	0.65509 (13)	0.41510 (4)	0.0533 (3)	
C5	−0.0214 (2)	0.85891 (16)	0.44174 (5)	0.0668 (4)	
H5A	−0.0084	0.9418	0.4381	0.080*	
C6	−0.0961 (2)	0.81261 (18)	0.47852 (5)	0.0702 (4)	
C2	−0.0617 (2)	0.61339 (16)	0.45340 (5)	0.0682 (4)	
H2A	−0.0753	0.5307	0.4576	0.082*	
C13	0.0497 (2)	0.68788 (15)	0.11415 (5)	0.0679 (4)	
H13A	0.0469	0.6822	0.0833	0.102*	
H13B	0.1360	0.7477	0.1226	0.102*	
H13C	−0.0667	0.7106	0.1246	0.102*	
C1	−0.1175 (2)	0.69190 (19)	0.48511 (5)	0.0755 (5)	
H1A	−0.1686	0.6631	0.5104	0.091*	

### Atomic displacement parameters (Å^2^)

**Table:**

	*U*^11^	*U*^22^	*U*^33^	*U*^12^	*U*^13^	*U*^23^
O2	0.0827 (8)	0.0466 (6)	0.0744 (7)	0.0011 (5)	−0.0083 (6)	0.0087 (5)
O1	0.0825 (8)	0.0703 (7)	0.0494 (6)	0.0061 (6)	−0.0002 (5)	−0.0128 (5)
C16	0.0457 (7)	0.0435 (7)	0.0600 (8)	−0.0068 (5)	−0.0021 (6)	0.0038 (6)
C9	0.0386 (6)	0.0389 (6)	0.0567 (7)	−0.0051 (5)	0.0001 (5)	−0.0038 (5)
C15	0.0590 (8)	0.0532 (8)	0.0578 (8)	−0.0064 (6)	−0.0015 (6)	0.0086 (6)
C10	0.0546 (7)	0.0400 (6)	0.0504 (7)	−0.0005 (5)	0.0041 (6)	−0.0071 (5)
C12	0.0474 (7)	0.0532 (8)	0.0508 (7)	−0.0031 (6)	0.0009 (6)	−0.0102 (6)
F	0.1113 (9)	0.1308 (10)	0.0624 (6)	0.0123 (7)	0.0144 (6)	−0.0209 (6)
C14	0.0613 (8)	0.0466 (7)	0.0525 (8)	−0.0033 (6)	−0.0038 (6)	0.0037 (6)
C11	0.0551 (8)	0.0425 (7)	0.0516 (7)	0.0011 (6)	0.0011 (6)	−0.0035 (5)
C4	0.0687 (9)	0.0642 (9)	0.0407 (7)	−0.0032 (7)	−0.0014 (6)	0.0075 (6)
C8	0.0549 (8)	0.0407 (7)	0.0693 (9)	0.0032 (6)	−0.0018 (6)	−0.0069 (6)
C7	0.0638 (9)	0.0497 (8)	0.0665 (9)	0.0064 (6)	0.0020 (7)	−0.0202 (7)
C3	0.0531 (7)	0.0624 (9)	0.0443 (7)	−0.0044 (6)	−0.0045 (6)	0.0098 (6)
C5	0.0815 (11)	0.0689 (10)	0.0501 (8)	0.0018 (8)	−0.0055 (7)	0.0013 (7)
C6	0.0671 (10)	0.0968 (13)	0.0467 (8)	0.0076 (9)	−0.0016 (7)	−0.0049 (8)
C2	0.0699 (10)	0.0777 (10)	0.0571 (9)	−0.0075 (8)	0.0018 (7)	0.0184 (8)
C13	0.0723 (10)	0.0804 (11)	0.0508 (8)	−0.0039 (8)	0.0007 (7)	−0.0011 (7)
C1	0.0704 (10)	0.1107 (15)	0.0454 (8)	−0.0035 (10)	0.0081 (7)	0.0135 (9)

### Geometric parameters (Å, °)

**Table:**

O2—C16	1.2334 (17)	C4—C5	1.377 (2)
O1—C12	1.3561 (18)	C4—C3	1.394 (2)
O1—C13	1.424 (2)	C4—H4A	0.9300
C16—C9	1.478 (2)	C8—C7	1.363 (2)
C16—C14	1.478 (2)	C8—H8A	0.9300
C9—C10	1.3892 (19)	C7—H7A	0.9300
C9—C8	1.4002 (19)	C3—C2	1.398 (2)
C15—C14	1.319 (2)	C5—C6	1.371 (2)
C15—C3	1.455 (2)	C5—H5A	0.9300
C15—H15A	0.9300	C6—C1	1.362 (3)
C10—C11	1.382 (2)	C2—C1	1.378 (3)
C10—H10A	0.9300	C2—H2A	0.9300
C12—C11	1.3864 (19)	C13—H13A	0.9600
C12—C7	1.391 (2)	C13—H13B	0.9600
F—C6	1.361 (2)	C13—H13C	0.9600
C14—H14A	0.9300	C1—H1A	0.9300
C11—H11A	0.9300		
			
C12—O1—C13	118.30 (11)	C7—C8—H8A	119.5
O2—C16—C9	120.57 (12)	C9—C8—H8A	119.5
O2—C16—C14	120.38 (13)	C8—C7—C12	120.62 (12)
C9—C16—C14	119.03 (12)	C8—C7—H7A	119.7
C10—C9—C8	117.65 (13)	C12—C7—H7A	119.7
C10—C9—C16	123.23 (12)	C4—C3—C2	117.37 (14)
C8—C9—C16	119.11 (12)	C4—C3—C15	122.98 (12)
C14—C15—C3	127.26 (14)	C2—C3—C15	119.63 (14)
C14—C15—H15A	116.4	C6—C5—C4	118.32 (16)
C3—C15—H15A	116.4	C6—C5—H5A	120.8
C11—C10—C9	121.91 (12)	C4—C5—H5A	120.8
C11—C10—H10A	119.0	F—C6—C1	118.87 (15)
C9—C10—H10A	119.0	F—C6—C5	118.44 (18)
O1—C12—C11	124.31 (13)	C1—C6—C5	122.70 (16)
O1—C12—C7	116.05 (12)	C1—C2—C3	121.55 (16)
C11—C12—C7	119.64 (13)	C1—C2—H2A	119.2
C15—C14—C16	122.03 (13)	C3—C2—H2A	119.2
C15—C14—H14A	119.0	O1—C13—H13A	109.5
C16—C14—H14A	119.0	O1—C13—H13B	109.5
C10—C11—C12	119.17 (13)	H13A—C13—H13B	109.5
C10—C11—H11A	120.4	O1—C13—H13C	109.5
C12—C11—H11A	120.4	H13A—C13—H13C	109.5
C5—C4—C3	121.62 (14)	H13B—C13—H13C	109.5
C5—C4—H4A	119.2	C6—C1—C2	118.45 (15)
C3—C4—H4A	119.2	C6—C1—H1A	120.8
C7—C8—C9	121.01 (13)	C2—C1—H1A	120.8

### Hydrogen-bond geometry (Å, °)

**Table:**

*D*—H···*A*	*D*—H	H···*A*	*D*···*A*	*D*—H···*A*
C11—H11A···O2^i^	0.93	2.50	3.376 (2)	158
C15—H15A···O2	0.93	2.50	2.825 (2)	101

Symmetry codes: (i) −*x*, *y*+1/2, −*z*+1/2.
